# Endometrial thickness and early pregnancy complications after frozen-thawed embryo transfers

**DOI:** 10.3389/fendo.2023.1066922

**Published:** 2023-08-21

**Authors:** Lin Song, Zhiqin Bu, Yingpu Sun

**Affiliations:** ^1^ Center for Reproductive Medicine, The First Affiliated Hospital of Zhengzhou University, Zhengzhou, China; ^2^ Henan Key Laboratory of Reproduction and Genetics, The First Affiliated Hospital of Zhengzhou University, Zhengzhou, China; ^3^ Henan Provincial Obstetrical and Gynecological Diseases (Reproductive Medicine) Clinical Research Center, The First Affiliated Hospital of Zhengzhou University, Zhengzhou, China; ^4^ Henan Engineering Laboratory of Preimplantation Genetic Diagnosis and Screening, The First Affiliated Hospital of Zhengzhou University, Zhengzhou, China

**Keywords:** endometrial thickness, frozen thawed embryo transfer, ectopic pregnancy, early spontaneous miscarriage, pregnancy complications

## Abstract

**Background:**

The relationship between endometrial thickness and pregnancy safety after *in vitro* fertilization treatment is an important topic that should provoke attention. The aim of this study was to demonstrate the relationship between endometrial thickness on day of embryo transfer and early pregnancy complications, including ectopic pregnancy and early miscarriage, in frozen thawed embryo transfer (FET) cycles.

**Methods:**

Patients undergoing their first FET cycles were included into this study from January 2010 to December 2021. Patients were divided into three groups according to endometrial thickness on day of embryo transfer: Thin, ≤ 7 mm; Medium, 7-14 mm; Thick, ≥ 14 mm. Ectopic pregnancy and early miscarriage were the two primary outcomes. Endometrial thickness was the main measured variable. The risk factors of these two compilations were determined based on univariate analysis and multivariate logistic regression analysis.

**Results:**

A total of 11138 clinical pregnancies were included. The overall ectopic pregnancy and early spontaneous miscarriage rates were 2.62% and 13.40%. The ectopic pregnancy and early spontaneous miscarriage rates were significantly higher in patients with thin endometrium as compared with those in the other two groups (ectopic pregnancy rate: 5.06% vs. 2.62% vs. 1.05%; P < 0.001; early spontaneous miscarriage rate: 15.18% vs. 13.45% vs. 11.53%; P < 0.001). In multivariate logistic regression analysis, thin endometrium was an independent factor to predict ectopic pregnancy [adjusted odds ratio (aOR): 5.62; 95% confidence interval (CI): 2.51–12.58, P < 0.001], and to predict early spontaneous miscarriage rate (aOR: 1.57; 95% CI: 1.21–1.74, P < 0.001).

**Conclusion:**

Thin endometrium on day of embryo transfer in FET cycles is an independent predictor for early pregnancy compilations, including ectopic pregnancy and early spontaneous miscarriage.

## Introduction

Frozen-thawed embryo transfer (FET), as one of the most important related technologies of *in vitro* fertilization (IVF) and embryo transfer, has been widely used currently, especially after the consistent increasing number of frozen embryos and awareness of ovarian hyper-stimulation syndrome. In fresh embryo transfer cycles after oocyte retrieval, there are several factors, including ovarian stimulation protocols, estrogen and progesterone levels before embryo transfer, and endometrial thickness those affect IVF outcomes and pregnancy complications. Compared to fresh embryo transfer cycles, FET has its own advantages in studying factors affect IVF pregnancy outcomes as many factors can be well controlled.

It is known that embryo quality and endometrial receptivity are two key elements for a successful pregnancy in IVF treatment. As for endometrial receptivity, there are non-invasive and invasive methods to assess it. Endometrial thickness assessment can be performed noninvasively, and has been used as a routine practice in IVF centers. Many previous studies demonstrated that endometrial thickness, which is measured either on day of progesterone administration or on day of embryo transfer, is positively associated with pregnancy outcomes, including clinical pregnancy rate and live birth rate (1, 2). In addition, recent studies also showed that the change of endometrial thickness (endometrium compaction) between day of embryo transfer and day of ovulation in FET cycles impacts pregnancy outcomes (3–5).

The relationship between endometrial thickness and IVF pregnancy rates has been extensively studied. However, the pregnancy safety after IVF treatment is another important topic that should provoke attention. Recently, a large cohort study with more than 10,000 pregnancies after embryo transfer showed that patients with an endometrial thickness > 7.6 mm had a significant lower risk of ectopic pregnancy compared to those with an endometrial thickness < 7.6 mm (6). In addition, data also showed that the incidence of hypertensive disorders of pregnancy in patients with a thin endometrial thickness (< 8 mm) was significantly greater than that in patients with endometrial thickness being 8 mm-12 mm (7). Moreover, the impact of thin endometrial thickness and other pregnancy complications, such as spontaneous miscarriage, preterm birth, and low birth weight has also been explored, but with controversial results (8–10).

As one of the largest IVF centers in China, our center has advantages in cohort study design as the convenience in high quality clinical data collection and management, and has gained wider experience in exploring the impact of endometrial thickness and IVF outcomes. The aim of this study was to demonstrate the relationship between endometrial thickness on day of embryo transfer and early pregnancy complications (ectopic pregnancy and early miscarriage) in FET cycles.

## Materials and methods

Institutional Review Board approval was not needed for this study, because all patients included underwent the routine clinical treatment in our center and no additional intervention or sampling was performed. Written informed consent was obtained from all patients before IVF treatment for physicians collecting basic information and treatment data. Data in this study were from the Clinical Reproductive Medicine Management System/Electronic Medical Record Cohort Database (CCRM/EMRCD) in Reproductive Medical Center, First Affiliated Hospital of Zhengzhou University.

Patients undergoing their first FET cycles were included this study from January 2010 to December 2021. Included first FET cycles were with complete information regarding basic clinical characteristics and treatment outcome. Exclusion criteria were as follows: uterine malformation; uterine adhesion, tuberculosis, and polyps; sperm/oocyte donation cycles, pre-implantation genetic testing cycles.

Endometrial thickness was measured in the morning on day of embryo transfer using trans-vaginal probe. During endometrial thickness assessment, the ultrasound image of the endometrium from the inner cervical canal to the uterus fundus was firstly displayed. The maximum distance between the myometrium and the endometrium on both sides was then measured. These steps were performed by well-trained physicians according to our standard operation procedure in our center. Endometrial patterns were classified based on the morphology of the endometrium, as follows: pattern A, triple-line or multi-layered type; pattern B, slight-triple-line type; and pattern C, even-and-strong-echoed. On day of embryo transfer, only endometrium with pattern C was included into this study.

Endometrium preparation protocols were natural cycles and estrogen-progesterone cycles. For natural cycles used for patients with regular menstruation, endometrial thickness and follicles were measured on menstrual day 7-9, with blood test of estrogen and progesterone levels if necessary. On day of ovulation, 40 mg progesterone in oil was used before embryo transfer. On day of embryo transfer, 10 mg of oral progesterone (Duphaston; Solvay Pharmaceuticals B.V., Veenendaal, The Netherlands) was used 3 times per day, combined with daily use of 90 mg of vaginal progesterone (Crinone, Merck Serono, Germany). Cleavage stage embryos and blastocyst were transferred 3, or 4 days after ovulation, respectively.

In estrogen-progesterone cycles, oral estradiol (1-2 mg [Progynova]; Bayer, Leverkusen, Germany) twice a day on cycle day 3 was firstly given. This dose was adjusted based on endometrial thickness 7 days later, and the maximum dose was 8 mg in total per day. After 12–18 days, if no leading follicle was present, 60 mg progesterone in oil and 10 mg of oral progesterone (Duphaston; Solvay Pharmaceuticals B.V., Veenendaal, The Netherlands) (this dose will be changed to 30 mg 3 days later) was added for endometrial transformation. Cleavage stage embryos and blastocyst were transferred 5, or 6 days after progesterone administration, respectively.

Patients were divided into three groups according to endometrial thickness: Thin, ≤ 7 mm; Medium, 7-14 mm; Thick, ≥ 14 mm. The diagnosis of ectopic pregnancy, and criteria for tubal infertility were elaborated in our previous studies (11). Clinical pregnancy was defined as a gestational sac or multiple sacs seen *via* ultrasound 5 weeks after embryo transfer with detection of cardiac activity. The definition of early spontaneous miscarriage was miscarriage that occurred before 12 weeks of gestation.

### Statistical analysis

Firstly, the rates of ectopic pregnancy and early spontaneous miscarriage were compared in different groups according to basic parameters (age, BMI, type of embryos transferred, endometrial thickness, etc) using Chi-square test. Parameters selection was based on our experiences and previous publications (8, 11). In addition, only those parameters were found to be possibly associated with ectopic pregnancy or early spontaneous miscarriage were included further for multivariate logistic regression analysis using SPSS (Statistical Package for Social Science, SPSS Inc, Chicago, IL) 21.0. A P value < 0.05 was considered statistically significant.

## Results

**Figure 1:**
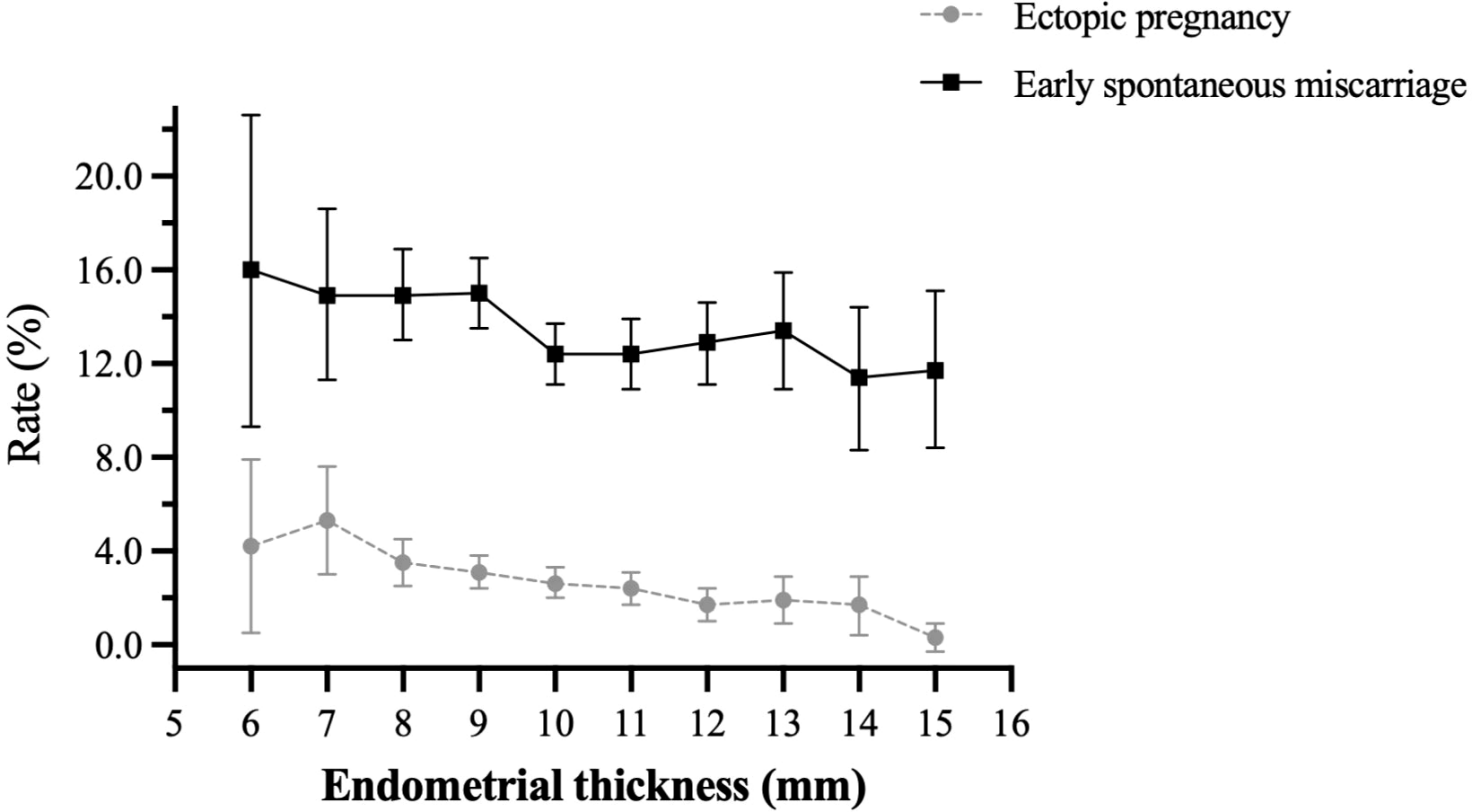
Overall ectopic pregnancy rate and early spontaneous miscarriage rate in patients with different endometrial thickness.

A total of 11138 clinical pregnancies were included. Each patient was included only once. The overall ectopic pregnancy and early spontaneous miscarriage rates were 2.62% (292/11138) and 13.40% (1492/11138), respectively. As shown in **Figure 1**, both ectopic pregnancy rate and early spontaneous miscarriage rate declined with increasing endometrial thickness.

**Table 1** shows the basic demographic information of patients with different endometrial thickness. BMI and basic FSH levels were similar in the three groups. However, the differences in patient age were statistically different (31.15 vs. 30.22 vs. 30.72, P < 0.001). In addition, except for the number of embryos transferred, which was comparable among the three groups, patients with a thin endometrium had a higher proportion of blastocyst transfer (59.72% vs. 47.93% vs. 44.04%, P < 0.001) and EP protocol for endometrium preparation (79.35% vs. 67.30% vs. 41.42%; P < 0.001).

**Table 1:** Basic characteristic in patients with different endometrial thickness.

	Thin (≤ 7 mm)	Medium (8-14 mm)	Thick (≥ 14 mm)	*P*
No. of cycles	494	9881	763	
Age (year)	31.15 ± 4.94	30.22 ± 4.57	30.72 ± 4.49	<0.001
Basic FSH (mIU/mL)	6.64 ± 2.88	6.91 ± 6.27	7.11 ± 6.94	0.432
BMI (Kg/m^2^)	23.06 ± 3.09	22.82 ± 3.25	22.84 ± 3.23	0.287
Infertility diagnosis				<0.001
Primary infertility	28.34% (140/494)	46.24% (4569/9881)	45.35% (346/763)	
Secondary infertility	71.66% (354/494)	53.76% (5312/9881)	54.65% (417/763)	
Stage of embryo				<0.001
Cleavage stage	40.28% (199/494)	52.07% (5145/9881)	55.96% (427/763)	
Blastocyst	59.72% (295/494)	47.93% (4736/9881)	44.04% (336/763)	
Protocols				<0.001
Natural cycle	20.65% (102/494)	32.70% (3231/9881)	58.58% (447/763)	
E-P	79.35% (392/494)	67.30% (6650/9881)	41.42% (316/763)	
No. of embryos transferred	1.68 ± 0.48	1.70 ± 0.46	1.69 ± 0.46	0.217

Values are the mean ± SD unless otherwise noted. FSH, follicle-stimulating hormone; BMI, body mass index; E-P, Estrogen-progesterone.

The ectopic pregnancy rate is shown in **Table 2**. It varied in patients with different types and numbers of embryos transferred and in those with or without tubal factor infertility. Moreover, the ectopic pregnancy rate in patients with thin, medium, and thick endometrium was significantly different, at 5.06% (25/494), 2.62% (259/9881), and 1.05% (8/763), respectively. Early spontaneous miscarriage rate was also higher in women with advanced age, those with a higher BMI, and those with blastocyst transfer. Similarly, it was also found that miscarriage rate was significantly higher in women with thin endometrium as compared with that in patients with thick endometrium (15.18% vs. 13.45% vs. 11.53%; P < 0.001). However, neither ectopic pregnancy nor early spontaneous miscarriage rate was found to be different in patients with natural cycles and EP protocols for endometrium preparation.

**Table 2:** Ectopic pregnancy and early spontaneous miscarriage rates in frozen-thawed transfer cycles.

	Ectopic pregnancy	*P*	Early spontaneous miscarriage	*P*
Age (year)				
< 38	2.68% (276/10280)	0.152	12.06% (1240/10280)	<0.001
≥ 38	1.86% (16/858)		29.37% (252/858)	
BMI (kg/m^2^)				
≤18	1.54% (7/455)	0.273	10.99% (50/455)	<0.001
18-24	2.59% (184/7091)		12.58% (892/7091)	
≥ 24	2.81% (101/3592)		15.31% (550/3592)	
Infertility diagnosis				
Primary infertility	2.35% (119/5055)	0.114	11.77% (595/5055)	<0.001
Secondary infertility	2.84% (173/6083)		14.75% (897/6083)	
Tubal factor existed				
Yes	4.08% (170/4170)	<0.001	12.71% (530/4170)	0.104
No	1.75% (122/6968)		13.81% (962/6968)	
Stage of embryo				
Cleavage stage	3.41% (197/5771)	<0.001	12.44% (718/5771)	<0.001
Blastocyst	1.77% (95/5367)		14.42% (774/5367)	
Protocols				
Natural cycle	2.57% (97/3780)	0.794	12.91% (488/3780)	0.288
E-P	2.65% (195/7358)		13.65% (1004/7358)	
No. of embryos transferred				
1	1.81% (61/3369)	<0.001	16.18% (545/3369)	<0.001
2	2.97% (231/7769)		12.19% (947/7769)	
Endometrial thickness (mm)				
Thin (≤ 8)	5.06% (25/494)	<0.001	15.18% (75/494)	<0.001
Medium (8–14)	2.62% (259/9881)		13.45% (1329/9881)	
Thick (≥ 14)	1.05% (8/763)		11.53% (88/763)	

BMI, body mass index; E-P, Estrogen-progesterone.

To control for confounding factors that affect early pregnancy complications, multivariate logistic regression analysis was also performed. As shown in **Table 3**, after adjusting for tubal factor infertility, number of embryos transferred, and stage of embryos transferred, a thin endometrium was still an independent factor to predict ectopic pregnancy [adjusted odds ratio (aOR): 5.62; 95% confidence interval (CI): 2.51–12.58, P < 0.001]. Interestingly, in multivariate logistic regression analysis, after controlling for female age, BMI, infertility diagnosis, and stage or number of embryos transferred, it was found that, apart from advanced female age or elevated BMI, thin endometrium was also a risk factor for early spontaneous miscarriage rate (aOR: 1.57; 95% CI: 1.21–1.74, P < 0.001).

**Table 3:** Factors associated with ectopic pregnancy and early spontaneous miscarriage rates by logistic regression analysis in frozen-thawed transfer cycles.

	Ectopic pregnancy		Early spontaneous miscarriage	
	Adjusted OR (95% CI)	*P*	Adjusted OR (95% CI)	*P*
Age (year)				
< 38 (Reference)	–		1.00	
≥ 38			2.94 (2.43-3.38)	<0.001
BMI (Kg/m^2^)				
≤18 (Reference)	–		1.00	
18-24			1.20 (0.93-1.72)	0.145
≥ 24			1.26 (1.06-1.34)	<0.001
Infertility diagnosis				
Primary infertility (Reference)	–		1.00	
Secondary infertility			1.09 (0.97-1.22)	0.134
Tubal factor existed				
No (Reference)	1.00		–	
Yes	2.41 (1.32-4.36)	<0.001		
Stage of embryo				
Blastocyst (Reference)	1.00		1.00	
Cleavage stage	1.91 (1.42-2.56)	<0.001	0.97 (0.84-1.11)	0.660
No. of embryos transferred				
1 (Reference)	1.00	0.483	1.00	
2	1.14(0.81-1.62)		0.78 (0.71-1.22)	0.182
Endometrial thickness (mm)				
Thick (≥ 14) (Reference)	1.00		1.00	
Medium (8–14)	2.60 (1.28-5.28)	<0.001	1.22 (0.91-1.78)	0.154
Thin (≤ 8)	5.62 (2.51-12.58)	<0.001	1.57 (1.21-1.74)	0.020

BMI, body mass index; OR, odds ratio; CI, confidence interval.

## Discussion

Infertility is a global health issue affecting millions of people of reproductive age worldwide. However, compared with infertility itself, pregnancy complications after IVF treatment are more frustrating, especially in ectopic pregnancy and early spontaneous miscarriage cases. As we mentioned earlier, the impact of endometrial thickness on late pregnancy outcomes, such as preterm birth, placenta previa, etc has been explored in a few studies (12, 13). However, these late complications are more complicated and are associated with many other factors that are out of our control, such as diet habit, infection of amniotic fluid or lower genital tract, and trauma.

In the current study, we mainly focused on the impact of endometrial thickness and early pregnancy complication from a large sample of patients, and found that thin endometrium was an independent risk factor for both ectopic pregnancy and early spontaneous miscarriage rates.

During IVF treatment, the occurrence of an ectopic pregnancy is not only a risk factor for recurrent ectopic pregnancy in the next pregnancy, but also brings financial and psychological burden to infertile couples (14). Currently, there was no consensus on the risk factors for ectopic pregnancy after IVF treatment, however, tubal factor infertility; high estrogen levels before embryo transfer seem to increase the incidence of ectopic pregnancy. On the contrast, blastocyst transfer was a protective factor as compared with cleavage stage embryo transfer (11, 15, 16).

As for the association between endometrial thickness and ectopic pregnancy, previous large data showed that a thin endometrium increased ectopic pregnancy rate in both fresh and FET cycles (17–19), which was consistent with results from ours. However, in Rombauts’s study, repeated cycles were presented (17). In the current study, we only included first IVF cycles, and made a thorough analysis of possible ectopic pregnancy related factors. Then, the next question was why thin endometrium increased ectopic pregnancy rate.

One of the possible explanations was that, the uterine peristalsis may change and subsequently affect pregnancy in thin endometrium patients. In 2014, Zhu et al. found that the uterine peristaltic wave frequency before embryo transfer was inversely related to the clinical pregnancy rates, and then a hypothesis was made that intense uterine peristalsis could extrude embryos out of the uterine cavity, leading to implantation failure or ectopic pregnancy (17, 20). Recently, Zhao et al. also suspected that a thin endometrial thickness could be linked with abnormal endometrial peristaltic waves or abnormal endometrial receptivity, and then resulting in ectopic pregnancy (21). Moreover, data also showed that controlled ovarian stimulation cycles with super-physiological estrogen significantly increased the uterine peristaltic waves (22), it was reasonable to found that ectopic pregnancy rate was higher in fresh embryo transfer cycles as compared with that in FET cycles (23); and higher in patients with elevated estrogen as compared with those with lower hormonal milieu (11, 24). In FET cycles, patients with thin endometrium tended to take more pills or other actions to increase estrogen levels and endometrial thickness, which eventually increased ectopic pregnancy rate as well. Secondly, other scholars also speculated that the higher status of oxygen concentration in a thin endometrium was similar with that in the fallopian tube. This could also increase the higher risk of ectopic pregnancy as higher oxygen concentrations inhibit the growth of embryos (25).

Early spontaneous miscarriage is another common pregnancy complication after IVF treatment. Currently, there are no precise data on the incidence of spontaneous miscarriage after IVF, but most studies indicate that it is higher than that in natural conceptions (26, 27).

Firstly, results from the current study were in line with previous ones, in which advanced female age and elevated BMI were risk factors with early spontaneous miscarriage (28–31). However, most previous studies mainly focused on the impact of endometrial thickness on clinical pregnancy or live birth rates, few ones took spontaneous miscarriage rate as a primary outcome. In addition, conflicting results existed in regard to the impact of endometrial thickness on spontaneous miscarriage rate (8, 32). In this large cohort study, data also showed that thin endometrial thickness was also an independent risk factor for early spontaneous miscarriage after adjusting female age and BMI.

It is known that decidualization is an essential prerequisite for implantation. During this process, stromal cells undergo remarkable morphogenetic and vascular changes under the influence of the steroid hormones. Thus, lacking of these receptors leads to a failure of pregnancy and miscarriage (33, 34). A recently study showed that thin endometrium was associated with reduced expression of estrogen receptor in stromal cells both during proliferative and secretory phase (35). This may explain the phenomenon that early spontaneous miscarriage elevated in thin endometrium patients as the existence of compromised decidualization.

One of the strengths in this study was the large sample from a single center. In addition, each patient was included only once. The homogeneity of patient’s data and physician standard operating procedure made the data more reliable. In addition, all patients underwent a detailed ultrasound examination to determine the endometrial thickness and morphology before embryo transfer in our center. Since only patients with Pattern C Endometrium were included, there was no effect of endometrial morphology on the miscarriage rate in this study. Moreover, all patients in this study underwent hysteroscopy examination before each cycle, and only those with normal uterine cavity were included. Severe uterine adhesion, uterine polyps, fibroids, and other lesions were excluded.

However, several limitations also existed. Not all bias could be controlled as the retrospective nature of this study. In patients with thin endometrium, it was obvious that the proportion of E-P cycles was much higher and patient basic demographic data were different. We performed multivariate logistic regression analysis to minimize these side effects. More importantly, hysteroscopy examination mainly detects the form of uterine cavity, the specific etiology for thin endometrium needed further extensive study.

In summary, data from this large cohort study show that, thin endometrium on day of embryo transfer in FET cycles is an independent predictor for early pregnancy compilations, including ectopic pregnancy and early spontaneous miscarriage. This reminds us to pay attention to the association between thin endometrial thickness and IVF safety. Although the treatment of thin endometrium is still a challenge, these data help us provide more comprehensive consultation and make appropriate decisions for patients with thin endometrium.

## Data availability statement

The original contributions presented in the study are included in the article/supplementary material. Further inquiries can be directed to the corresponding author.

## Ethics statement

The studies involving human participants were reviewed and approved by Institutional Review Board (IRB) of First Affiliated Hospital of Zhengzhou University. The patients/participants provided their written informed consent to participate in this study.

## Author contributions

LS and ZB contributed to the conception, design, acquisition and interpretation of data, and drafting of the manuscript. YS supervised the study. All authors contributed to the article and approved the submitted version.
